# Effect of different protective agents on enamel erosion: An in vitro investigation

**DOI:** 10.4317/jced.55278

**Published:** 2019-02-01

**Authors:** Marco Colombo, Alberto Dagna, Giulia Moroni, Marco Chiesa, Claudio Poggio, Giampiero Pietrocola

**Affiliations:** 1 Department of Clinical-Surgical, Diagnostic and Pediatric Sciences, Section of Dentistry, University of Pavia, Pavia, Italy; 2 Department of Molecular Medicine, Unit of Biochemistry, University of Pavia, Pavia, Italy

## Abstract

**Background:**

The purpose of this *in vitro*study was to compare the effect of different protective agents on enamel erosion by measuring mean percentage weight loss.

**Material and Methods:**

Extracted teeth were sectioned into uniform slabs and enamel specimens were randomly distributed to different groups. Initial weight of all enamel specimens was registered. The protective agents used in this study were Tooth Mousse, MI Paste Plus, Remin Pro and Remin Pro Forte. A control group was treated just with tap water. All the specimens were immersed in Coca-Cola for a total of 8 min at room temperature, dried and weighed. Enamel dissolution caused by acidic soft drink was analyzed: specimens were weighed after each immersion period and mean percent weight loss was calculated. Weight loss data were subjected to Analysis of Variance (One-way ANOVA) followed by Bonferroni’s post hoc tests.

**Results:**

All the groups showed a statistically significant loss of weight (*p*<0.01) during the testing periods, increased after 8 days (~55%) and 12 days (~70%) of exposure. Specimens treated with protective agents showed significantly lower % of weight loss especially with Remin Pro or Remin Pro Forte.

**Conclusions:**

Soft drinks can cause enamel erosion, but protective agents tested may enhance enamel resistance against erosion.

** Key words:**Enamel, erosion, protective agents, soft drinks, toothpastes.

## Introduction

Dental erosion is a process involving the dissolution of enamel and dentine by non-bacterial acids that soften the enamel surface, so mechanical factors such as abrasion and attrition may result in further loss of tooth tissue (1). The prevalence of dental erosion is thought to be increasing, due to the wide availability and frequent consumption of acidic drinks such as soft drinks, sports drinks and fruit juices (2). The development of erosion involves a chemical process in which the inorganic phase of the tooth is demineralized, thereby reducing the hardness of tooth substrates (3). Typical acid sources come from the diet, medications, occupational exposure, and lifestyle activities (4,5).

Many strategies have been established to prevent dental erosion (6). Toothpastes were considered effective and affordable vehicles to enhance enamel and dentin resistance (7) and the incorporation of protective agents in toothpastes has become increasingly common. Dental sensitivity is a problem often related to acid erosion and a common complaint among patients. Currently, conventional fluoride-based toothpastes do not seem to be able to effectively protect teeth against erosion (8). Recently, new toothpastes formulations have been introduced to contrast enamel and dentin erosion (9). Among the large amount of commercially available products, several new toothpaste technologies were subject of our previous studies (10,11). The casein phosphopeptides amorphous calcium phosphate CPP-ACP solutions have also been shown to significantly remineralize enamel subsurface lesions *in vitro* (12). CPP-ACP has been successfully incorporated into oral health products such as mouthrinses, sugar-free chewing gums and sports drinks to reduce enamel erosion (13). It has been suggested that casein phosphopeptide-amorphous calcium phosphate with fluoride (CPP-ACPF) provide additional fluoride along with calcium and phosphate ions for remineralization of enamel (14).

Fluoride and stannous ions have shown anti-erosive action *in vitro* and *in situ* (15). Topical application of fluoridated rinses (112–450 ppm F, as NaF) may protect against erosive tooth wear (16) by forming a CaF2 or CaF2-like layer on the enamel surface. Similarly, stannous ion-containing rinses (800 ppm Sn, as SnF2 or SnCl2) seem to prevent dental erosion by depositing a stable acid-resistant layer on the tooth surfaces (17,18). Sn-containing rinses may also react with dental hard tissues, due to the low pH, leading to the incorporation of the Sn ion into the enamel, thereby creating a more acid-resistant substrate (19).

The purpose of this *in vitro* study was to compare the effect of different protective agents on enamel erosion, measuring mean percentage weight loss before and after acidic beverage exposure.

## Material and Methods

Freshly extracted and sound human permanent incisors were used for this study. Inclusion criteria were: no hypo calcification, no caries, no macroscopic fractures. The teeth were carefully cleaned from calculus and debris and stored in a 1% Chloramine-T solution (Fisher Chemical, Fair lawn, NJ, USA) consisting of 12% active chlorine diluted in distilled water. The crown of each tooth was removed at cementum-enamel junction utilizing a straight fissure carbide bur mounted on a high-speed water-cooled handpiece. One transversal section of 2-mm thickness was obtained from the facial surface of each crown using a pre-programmed automatic Accutom-50 diamond cutter (Accutom Hard Tissue Microtome, Struers, Ballerup, Denmark). Each slice was then sectioned in two sections, obtaining a total of two samples. Enamel specimens were catalogued and stored into distilled water at room temperature.

Initial weight of each enamel specimen was registered: all specimens were dried on blotting paper at room temperature for one hour and weighed using a precision balance (Mettler-Toledo, model AE1633, Novate Milanese, Italy, metering accuracy 0.01 mg).

The samples were randomly attributed to 5 groups (n= 10). Specimens of group 1 were treated only with tap water (control). Tap water (pH 7.20) was chosen as a negative control because it was expected to have no or minimal demineralization effect on enamel.

**Figure 1:**
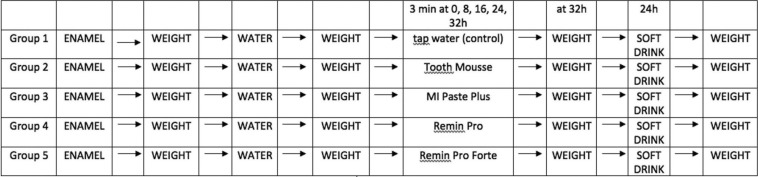
Diagram of the study design.

Specimens of groups 2, 3, 4, 5 were treated with application of protective pastes onto the surface without brushing for 3 min at 0, 8, 24, 32 h (Fig. 1). Four different protective pastes (Tooth Mousse, MI Paste Plus, Remin Pro and Remin Pro Forte) were evaluated. The characteristics, chemical composition and manufacturer of the tested products are reported in  Table 1. All the treated samples were weighed using the same precision balance and no statistically significant differences in term of weight variation before or after protective pastes application were found (*p*>0.05, ANOVA with Bonferroni post hoc test).

**Table 1:**
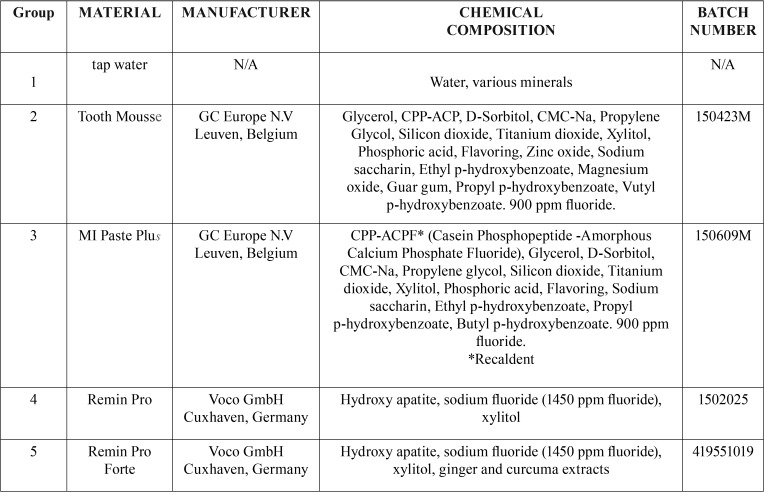
Protective materials used in this study.

Thereafter, all specimens were simultaneously placed in a PVC pannier suspended in a plastic container with 6 ml of a soft drink (Coca-Cola, The Coca-Cola Company, Milano, Italy) and immersed for 2 min at room temperature, then rinsed with deionized water. Four consecutive intervals of immersion procedure were carried out for a total of 8 minutes (20). Each specimen was removed from the soft drink using tweezers, dried with blotting paper, left at room temperature to dry for 60 minutes, and weighed. The mass loss was calculated as a percentage of the results obtained before the application of protective pastes or tap water (mass set to 0%). Continuous data were expressed as means and standard deviations. Weight loss data were subjected to Analysis of Variance (One-way ANOVA) followed by Bonferroni’s post hoc tests. Analyses were performed using Prism 4.0 (GraphPad). Two-tailed *P* values of 0.05 were considered statistically signﬁcant.

## Results

**Figure 2:**
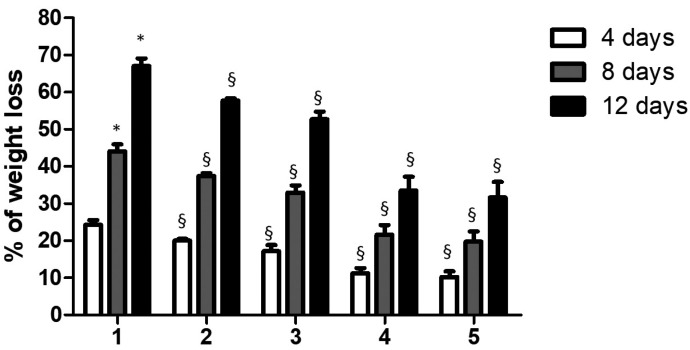
Relation between weight loss of enamel teeth specimens and time. The mass loss was calculated as a percentage of that observed prior the protective pastes or water (control) application (mass set to 0%). The reported data are the mean values (+/- SD). Symbol (*) indicates statistically significant differences (*p* < 0.01) in group 1 as determined by repeated-measures one-way ANOVA with a Bonferroni’s post hoc tests. Symbol (§) indicates statistically significant differences (*p* < 0.01) between group 1 and towards each other group as determined by repeated-measures one-way ANOVA with a Bonferroni’s post hoc tests.

Results are shown in Fig. 2 and in  Table 2. The acidic soft drink Coca-Cola caused ~25% of weight loss of enamel after 4 days of exposure. The weight loss of enamel significantly increased of ~ 55% and ~ 70% after 8 and 12 days of exposure respectively.

**Table 2:**
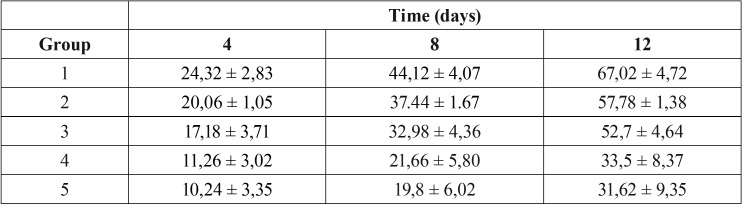
Mean (± SD) of the enamel weight loss for different groups. The mass loss was calculated as a percentage of that observed prior the protective pastes or water (control) application (mass set to 0%).

As shown in Fig. 2, Tooth Mousse and MI Paste Plus showed a similar trend in terms of weight loss of enamel. In both cases and for all the exposure times to Coca-Cola evaluated, the % of weight loss was significantly lower than that observed for control group (*P*<0.01). For all the tested times, specimens treated with Remin Pro or Remin Pro Forte, showed significantly lower % of weight loss compared to the control group (*P*< 0.01) or both groups 2 and 3 (*P*<0.01).

## Discussion

Surface roughness increase of enamel due to exposure to non-alcoholic soft drinks such as Coca-Cola has been well documented (21,22). This study aimed to explore if exposure to Coca-Cola after the treatment with protective agents caused the same loss of enamel, in order to evaluate if protective pastes have preventive effect on enamel erosion.

As previously stated, several factors play important roles in the potential destruction of tooth structure following exposure to soft drinks and to other sweetened beverages (23). Salivary pH lies within a range of 5.5 to 6.5, with a pH of 5.5 or below, accepted as a threshold level for destruction of tooth structure, i.e. caries and erosion (24). A sustained low salivary pH (<5.5) has been shown to be a result of intake of carbohydrates or sugars (sucrose, fructose), and acids (phosphoric, citric, and other organic acids) (25). These ingredients decrease the buffering capacity (of saliva) and maintain the oral pH below the threshold level (5.5 pH) necessary for alteration of enamel (26). A constant assault by acids or incorrect drinking habits can result in accelerated demineralization of the enamel (and dentin) surface (27). Phosphoric, citric acid and/or citrates found in many soft drinks are added as flavoring agents or acidulants, and can concurrently chelate to calcium, promoting a decreased buffering effect of saliva and thus increased tooth destruction (23,24). Presence of calcium, phosphorous and fluoride in soft drinks or fruit juices has been shown to be limiting factor of the erosion potential in the oral cavity (28,29).

According to Hemingway *et al.*, (30) with the higher concentration of calcium ions found in soft drinks, the less likely the enamel surface calcium ions will be detached. Another study by Low *et al.* (31) found a direct relationship between the weight loss of tooth enamel and loss of calcium ions. Still yet another study by Davis *et al.* (32) has indicated that the erosive potential occurs in the first few minutes following exposure and is a factor of the beverage pH. Other investigations(32-34). evaluating the erosive potential of various beverages on permanent tooth structure revealed that Coca-Cola caused increased percent weight loss of the enamel specimens compared to Diet Coke. This finding suggests that beverages such as Coca-Cola, supplemented with refined carbohydrates or sugars (sucrose, high fructose corn syrup), compared to artificial sweeteners found in diet versions of the same beverage, may be contributing factors to tooth dissolution.

The results of the present study suggest the potential harmful effects of the tested beverage could be especially pertinent to persons with systemic conditions (Sjögrens syndrome) or to athletes, whereby salivary flow is reduced or non-existent causing xerostomia or dry-mouth conditions, because *in vitro* conditions of the oral environment cannot be replicated (35). Human saliva contains hundreds of proteins that serve as protective factors. Saliva also serves as a buffering agent, diluent, and repository of calcium and phosphate for remineralization - limiting the erosive potential associated with so drink consumption (36,37).

The characteristics, chemical composition and manufacturer of the protective agents tested (Tooth Mousse, MI Paste Plus, Remin Pro, Remin Pro Forte) are reported in  Table 1. Remin Pro and Remin Pro Forte are toothpastes containing Hydroxyapatite, sodium fluoride (1450 ppm fluoride) and xylitol. Tooth Mousse and MI Paste Plus are remineralizing agents based on casein phosphopeptide-stabilized amorphous calcium phosphate complexes (CPP-ACP) and casein phosphopeptide-stabilized amorphous calcium fluoride phosphate complexes (CPP-ACFP). The ability of casein to stabilize calcium and phosphate ions resides in sequences that can be released as small peptides (case in phosphopeptides) by partial enzymic digestion. The CPP-ACP and CPP-ACFP complexes have been incorporated into dental creams and stabilize or deliver bioavailable calcium, phosphate and fluoride ions (14). The softened enamel caused by soft drink, which represented the early stage of erosion, became hardened after four application of a CPP-ACP paste (20).

A significant challenge was the differing composition and concentrations of fluoride in the pastes. The results of this *in vitro* model demonstrated that the highly concentrated fluoride agents may protect the enamel. Remin Pro (1450 ppm) and in Remin Pro Forte (1450 ppm) have the highest concentration of fluoride, whilst MI Paste Plus (900 ppm) and Tooth Mousse (0 ppm) have considerably less.

Percent weight loss of specimens exposed to early stages in Coca-Cola showed linear progression with time. Remin Pro and Remin Pro Forte recorded the lowest values of weight loss after immersion in Coca Cola. This means that these pastes make the enamel resistant to acid attack effectively. Compared to the control group 1, the results of the specimens treated with all protective paste are statistically signficant (*p* < 0.01). For all the tested times, specimens treated with Remin Pro or Remin Pro Forte, showed significantly loss of weight compared to the other groups, maybe thanks to their high concentration in fluoride.

## Conclusions

Within the limitations of this *in vitro* study this study, all tested protective agents showed preventive effect against acidic action of soft drinks. Remin Pro and Remin Pro Forte showed better results than Tooth Mousse and MI Paste Plus. This difference in enamel protection is due to the different concentration of fluoride, which shows that fluoride can afford protection of enamel against erosion.

This study indicated that enamel is susceptible to erosion of soft drinks, but protective toothpaste can defend it against acidic erosion in different way according to their composition.
